# Invited Papers 1

**DOI:** 10.1038/sj.bjc.6601924

**Published:** 2004-06-22


British Journal of Cancer (2004) 91(Suppl 1), S1 – S2
© 2004 Cancer Research UK All rights reserved 0007 – 0920/04 $30.00
www.bjcancer.com

### INVITED PAPERS 1

### CT1

### MINIMAL INITIAL CHEMOTHERAPY PLUS INVOLVED FIELD RADIOTHERAPY (RT) VERSUS MANTLE FIELD RT FOR CLINICAL STAGE IA/IIA SUPRA-DIAPHRAGMATIC HODGKIN'S DISEASE (HD). RESULTS OF THE UK LYMPHOMA GROUP LY07 TRIAL.

Radford JA[1], Williams MV[2], Hancock BW[3], Hoskin P[4], Sun-Mynt H[5], Smith P[6], Qian W[6] and Linch D[7] on behalf of all LY07 collaborators.
[1]Christie Hospital, Manchester, [2]Addenbrookes Hospital, Cambridge, [3]Weston Park Hospital, Sheffield, [4]Mount Vernon Hospital, Northwood, [5]Clatterbridge Hospital, Wirral, [6]Cancer Research UK and University College Trials Centre and [7]University College Hospital, London.

Between November 1996 and June 2001, 226 pts with clinical stage I/II supra-diaphragmatic HD (no B symptoms or mediastinal bulk) were randomised to receive either mantle field RT (arm A, n=115) or 4 wks of VAPEC-B chemotherapy (doxorubicin 35mg/m² iv at wks 1 and 3, cyclophosphamide 350mg/m² iv at wk 1, etoposide 100mg/m² po days 1-5 at wk 3, vincristine 1.4mg/m² iv at wks 2 and 4 and bleomycin 10,000 IU/m² iv at wks 2 and 4 with prednisolone 50mg daily for 4 wks and prophylactic cotrimoxazole/ketoconazole) followed by involved field RT (arm B, n=111). In both arms RT dose was 30-40 Gy in daily fractions of 1.8-2 Gy.

At completion of treatment CR/CRu had been achieved by 91% pts in arm A and 90% in arm B, and PR by 7% in arm A and 9% in arm B. After a median follow-up of 51 months (range 0-85 months), 5 year relapse-free survival is 70% in arm A and 87% in arm B (p=0.002) and 5 year survival is 92% in arm A and 98% in arm B (p=0.036).

This analysis of the LY07 trial shows that 4 wks of VAPEC-B chemotherapy followed by involved field RT is superior to mantle field RT alone in early stage HD. These results are similar to those from an earlier pilot study in which mantle field RT was employed in both arms of the trial for pts with mediastinal involvement (abstract 070, 8th International Conference on Malignant Lymphoma, Lugano, 12-15 June 2002) and suggest that wide-field RT can be avoided in this group of pts by incorporating minimal initial chemotherapy into the treatment programme.

### CT2

### RANDOMISED CONTROLLED TRIAL OF ABVD VS. TWO MULTI-DRUG REGIMENS (MDRS) FOR ADVANCED HODGKIN'S LYMPHOMA (HL): INITIAL RESULTS OF UKLG LY09 (ISRCTN97144519).

B Hancock[1], J Radford[2], MH Cullen[3], J Walewski[4], MR Sydes[5], D Ryder[2], P Smith[6], S Clawson[5], SP Stenning[5], PM Johnson[7].
[1]Weston Park Hospital, Sheffield, UK; [2]Christie Hospital, Manchester, UK; [3]Queen Elizabeth Hospital, Birmingham, UK; [4] MSCM Cancer Centre, Warsaw, Poland; [5]MRC Clinical Trials Unit, London, UK; [7]BNLI, Cancer Research UK Clinical Trials Centre, London, UK; [7] Southampton General Hospital UK.

**Background:** This UKLG trial compared ABVD with two MDRs for treating advanced HL in terms of progression-free survival (PFS) and overall survival (OS). **Methods:** Patients with advanced (stage $II_{AX}$-IV) HL were randomised between standard ABVD (C) and each clinician's choice of two MDRs: Alternating ChlVPP/PABlOE (Alt) or Hybrid ChlVPP/EVA (Hyb). Six cycles were planned, plus two extra for slow responders, with guidance on radiotherapy (RT). MDR choice was specified at randomisation: patients (pts) were effectively randomised between $C_{Alt}$ and Alt or between $C_H$ and Hyb. **Results:** 807 pts were randomised Apr97 - Sept01; 406 allocated ABVD and 401 MDRs. The groups were well-balanced; 42% female, mean age 37 years, 36% IPI score 0-1. At 3yr median follow-up, 191 PFS events are reported. For the primary C vs MDRs comparison, the PFS hazard ratio (HR) = 1.00, 95% CI (0.76, 1.34), p=0.99 (HR > 1.0 favours C) with some evidence of heterogeneity according to choice of MDRs ($\chi^2$ interaction test, adjusting for IPI score, p=0.05). The survival HR = 1.25 (0.82, 1.90), p=0.33 with no clear evidence of heterogeneity according to choice of MDR ($\chi^2$ p=0.61). **Conclusion:** No clear evidence of a difference between ABVD and the MDRs was demonstrated with respect to PFS and OS, although preliminary analysis suggests the two MDRs may differ in efficacy. A centre effect was evident in the duration of chemotherapy and use of G-CSF and radiotherapy: this was related to the pre-randomisation choice of MDR but was balanced across the allocated treatment arms.

### CT3

### RESULTS FROM THE UK NCRI ADJUVANT BREAST CANCER (ABC) INTERNATIONAL TRIAL: POLYCHEMOTHERAPY AND OVARIAN ABLATION IN WOMEN WITH EARLY BREAST CANCER PRESCRIBED 5 YEARS TAMOXIFEN.

A M Brunt, JM Bliss, L Johnson, D Lawrence, J Yarnold, on behalf of the ABC Trial Management Group.

**Background:** The ABC trial tested the added benefits of ovarian ablation (OA) and/or chemotherapy (CT) in women with early breast cancer prescribed tamoxifen (T) 20mg daily for 5 years. **Methods:** Premenopausal women were randomised to OA (any type) vs. no OA (elective CT was allowed). Pre- and post-menopausal women in whom the added benefits of CT were unclear were randomised to CT (any type) vs. no CT (elective OA was allowed in premenopausal women). All patients had stage 1-3a breast cancer. Main endpoints: relapse-free survival (RFS) and overall survival (OS). **Results:** 1993 to 2000, 3854 patients were randomised from 106 UK and 16 non-UK centres. 1991 (987 CT, 1004 no CT) in the CT comparison; 2144 (1063 OA, 1081 no OA) in the OA comparison. **CT:** CT suggested an improvement in RFS (HR 0.86 [95%CI 0.73-1.01] p=0.06) and OS (HR 0.87 [95%CI 0.73-1.04] p=0.12). Adjusting for nodal status, ER, age and OA suggested a slightly increased the magnitude of benefit of CT (RFS: HR 0.83 (0.71-0.97) p=0.024, OS: HR0.84 (0.70-1.00) p=0.051). RFS was independent of ER status but not age. **OA:** With OA, no difference was seen in RFS (HR 0.98, 95%CI 0.83-1.16, p=0.81) or OS (HR 0.96, 95%CI 0.79-1.16, p=0.64), unadjusted, adjusted or in subgroups based on age, nodal status, ER status or elective CT. **Conclusion:** This is the first study to report the added impact of adjuvant CT or OA against a background of prolonged (5 years) T. Benefit of CT on RFS and OS is retained in this context but OA appears to have no additional effect.

### CT4

### DEVELOPMENT OF BONE METASTASES FROM PROSTATE CANCER: FIRST RESULTS OF THE MRC PR04 TRIAL (ISCRTN61384873)

Mason MD[1] and MRC PR04 collaborators; [1]University of Wales College of Medicine, Cardiff, UK.

**Background:** The skeleton is the most common site of metastases from prostate cancer. Bisphosphonates have been shown to slow development of metastases from breast cancer and myeloma and to modify pain from bone metastases from prostate cancer.

**Methods:** Phase III double-blind placebo-controlled randomised trial of oral bisphosphonate in men receiving standard treatment for locally advanced PCa with no evidence of bone metastases and WHO performance status 0-2. The primary endpoint was time to development of symptomatic bone metastases or PCa death. Treatment consisted of either 4 tablets/day oral sodium clodronate (2,080mg Loron 520) (A) or 4 tablets/day of matching placebo control (C). Patients were encouraged to stay on trial medication for 5 years or until the primary trial endpoint had been reached.

**Results:** **Patients:** 508 patients were randomised (target 500) over 3.5yrs (Jun94-Dec97): 254A, 254C. Baseline characteristics were well balanced. All patients have completed their allocated trial medication. **Efficacy and toxicity:** First results are due for first analysis in Spring 2004, based on at least 145 deaths with median follow-up of at least 5 years in surviving patients. Data will be presented by unblinded treatment arm in terms of trial medication (time on trial medication and reason for stopping trial),adverse events (rates of adverse events and dose-modifying adverse events, progression-free and overall survival (with numbers of events, log-rank test of treatment effect, median time to events, KM plots of time to events). **Conclusion:** The results will be considered in the context of sister trial, PR05, in metastatic disease which were published in JNCI in September 2003.

### CT6

**RANDOMISED TRIAL OF HIGH DOSE RAPID SCHEDULE WITH CONVENTIONAL SCHEDULE FOR STAGE 4 NEUROBLASTOMA OVER THE AGE OF ONE YEAR**

Pearson ADJ, Pinkerton CR, Lewis I, Imeson J, Ellershaw C, Machin D on behalf of the European Neuroblastoma Study Group and the United Kingdom Children's Cancer Study Group

This trial aimed to test whether increasing the dose intensity of induction chemotherapy, by rapid drug scheduling, in stage 4 neuroblastoma over one year will improve event-free survival (EFS). Eligible patients, were randomised to either COJEC (Rapid) or OPEC/OJEC (Standard). Each regimen utilised the same total cumulative dose of cisplatin, carboplatin, etoposide, cyclophosphamide and vincristine, but the dose intensity (mg/m2/week) with R was 1.8 fold higher. The S regimen was given every 21 days if there was haematological recovery while R every 10 days regardless of blood counts. In those patients who were responding, surgical excision of the primary tumour was attempted followed by myeloablative therapy (melphalan at 200 mg/m2) and haemopoietic stem cell rescue.

In total 262 patients: 132 S and 130 R were randomised. Of these 76.5% with S and 81.5% with R received at least 90% of the scheduled therapy and the relative dose intensity achieved was 1.85. Myleoablative therapy was delivered at a median of 53 days earlier with R. Apart from ototoxicity, the toxicity was tended to be greater with R. The 3 and 5 year EFS were 30.8% and 30%, respectively with R, compared to 24.2% and 18% with S.

In conclusion dose intensity can be increased in high-risk neuroblastoma, but there is little advantage in EFS and OS in the first two years after treatment. Beyond this time there is a suggestion of better outcome. The rapid induction regimen, COJEC, allows myeloablative intensity therapy to be given much earlier, which could contribute to outcome.

© 2004 Cancer Research UK

